# Circular RNA circENTPD7 suppresses the accumulation of PTEN to promote cell proliferation in non-small cell lung cancer

**DOI:** 10.1590/1678-4685-GMB-2022-0023

**Published:** 2022-08-19

**Authors:** Hongwei Yu, Yibin Zhang, Lu Zhang, Rufei Yang, Zhiwei Liao, Tongchong Zhou

**Affiliations:** 1 Affiliated Cancer Hospital & Institute of Guangzhou Medical University, Department of Radiotherapy, Guangzhou City, P.R. China.

**Keywords:** circENTPD7, NSCLC, PTEN, proliferation, cell cycle

## Abstract

The oncogenic role of circular RNA ENTPD7 (circENTPD7) in cancer biology has been reported in glioblastoma, while its role in non-small cell lung cancer (NSCLC) is unknown. This study was performed to investigate the involvement of circENTPD7 in NSCLC. NSCLC tissues and paired non-tumor tissues were collected from 64 NSCLC patients and the expression of circENTPD7 and PTEN were determined by RT-qPCR. Expression levels of PTEN protein in these tissue samples were measured by ELISA. The 64 NSCLC patients were subjected to a follow-up study to explore the role of circENTPD7 in predicting the survival of NSCLC. Overexpression of circENTPD7 was achieved in NSCLC cells, and the effects of overexpression of circENTPD7 on the expression of PTEN were measured by RT-qPCR and Western blot at mRNA and protein level, respectively. Cell proliferation was assessed by CCK-8 assay. CircENTPD7 was upregulated in NSCLC and high expression levels of circENTPD7 predicts the poor survival rate of NSCLC cells. In NSCLC tissues, circENTPD7 was inversely correlated with PTEN protein but not mRNA. In NSCLC tissues, overexpression of circENTPD7 resulted in downregulation of PTEN, but did not alter the expression of PTEN mRNA. Cell proliferation analysis showed that overexpression of circENTPD7 promoted the proliferation of NSCLC cells and reduced the inhibitory effects of overexpression of PTEN on cell proliferation. CircENTPD7 may suppress the accumulation of PTEN to promote cell proliferation in NSCLC.

## Introduction

Lung cancer is the most common type of malignancies for both incidence and mortality worldwide (Dela Cruz *et al.,* 2011). It is estimated that lung cancer affects 59.9 cases per 100,000 people and causes 44.7 deaths per 100,000 people in 2018 alone (Barta *et al.,* 2019). Even with the advances in the treatment of lung cancer, effective treatment approaches remain lacking, especially for the metastatic lung cancer cases, leading to poor survival (Zappa and Mousa, 2016; Hirsch *et al.,* 2017). Smoking is closely related to lung cancer (Rahal *et al.,* 2017). However, not all smokers develop lung cancer and lung cancer also affects never-smokers (Korpanty *et al.,* 2018; Zhou and Zhou, 2018), suggesting that other factors are also involved in lung cancer. In effect, lung cancer also requires the involvement of multiple molecular pathways (Cooper *et al.,* 2013). With the enhanced understanding of the pathogenesis of lung cancer, novel therapies that aim to treat lung cancer by regulating the expression of cancer-related genes have been developed (Janku *et al.,* 2010; Chan and Hughes, 2015). However, effective and safe targets for targeted therapy of lung cancer remain lacking. Circular RNAs are covalently closed single-strand RNAs that participate in cancer biology by regulating protein synthesis (Vo *et al.,* 2019). It has been well established that circRNAs can sponge one or multiple miRNAs through multiple miRNA binding sites to regulate the function of miRNAs, thereby regulating downstream protein synthesis (Vo *et al.,* 2019). Therefore, regulating the expression of certain circRNAs may indirectly regulating the expression of multiple cancer-related genes, suggesting the potential role of circRNAs as targets for cancer therapy (Lei *et al.,* 2019). However, functions of most circular RNAs in different types of cancer are unclear. Circular RNA circENTPD7 has been characterized as an oncogene in glioblastoma (Zhu *et al.,* 2020). Our preliminary analysis revealed the upregulated expression of circENTPD7 in non-small cell lung cancer (NSCLC), and its inverse correlation to PTEN protein. PTEN plays a role as tumor suppressor by antagonizing the conserved PI3K/AKT anti-apoptotic pathway, thereby suppressing cell survival in cancer biology (Chalhoub and Baker, 2009). Therefore, circENTPD7 may interact with PTEN to indirectly regulate PI3K/AKT pathway, thereby participating in NSCLC. This study aimed to explore the role of circENTPD7 in NSCLC, with a focus on its interaction with PTEN.

## Material and Methods

### NSCLC patients and follow-up

A total of 64 newly diagnosed NSCLC patients (40 males and 24 females, 30 cases of LUSC and 34 cases of LUAD) admitted at Affiliated Cancer Hospital & Institute of Guangzhou Medical University were enrolled in this study. The Ethics Committee of this hospital approved this study (Approval No. GMU26323). All patients signed the informed consent. Age of these patients ranged from 48 to 68 years old (mean age 57.6 ± 5.8 years old). The 64 patients were classified into AJCC stage I or II (n = 27) and III or IV (n = 37). Patients were treated with surgery, chemotherapy, radiation therapy or the combination of these therapies according to patients’ clinical stage and health conditions. Patients were visited every month for a total of 5 years. Patients’ clinical data was presented in Table 1.

**Table 1-:** Patients’ clinical data.

Clinicopathologic features	No.
Gender	
Male	40
Female	24
Age (years)	
＜60	34
≥60	30
Smoking status	
No-smoking	5
Smoking	59
AJCC stage	
I-II	27
III-IV	37
Subtypes	
LUSC	30
LUAD	34
Differentiation grade	
Well/moderately	19
Poorly/undifferentiated	45

### NSCLC tissue acquisition

NSCLC and paired normal tissues were collected from the 64 NSCLC patients through fine needle aspiration. Histopathological analysis confirmed all tissue specimens. Tissues were stored in liquid nitrogen before subsequent experiments.

### NSCLC cells and transient transfections

Human NSCLC cell lines NCI-H1703 (LUSC) and HCC4006 (LUAD) (ATCC, USA) were used. EMEM (10% FBS) was used as the cell culture medium. Cells were cultivated at 37 °C following instructions from ATCC. Vectors expressing circENTPD7 or PTEN were constructed with pcDNA3.1 (routine pcDNA3.1 and circular RNA pcDNA3.1, Invitrogen) as the backbone. To overexpress circENTPD7 or PTEN, cells (10^8^) were transfected with 1 μg circENTPD7 or PTEN expression vector using Lipofectamine 2000 (Invitrogen). Transfection with empty vector was used as NC group. Control (C) cells in both experiments were cells without transfections. The subsequent experiments were performed after cells were cultivated in fresh medium for another 48 h.

### RNA preparation and RT-qPCRs

Total RNAs were isolated from *in vitro* cultivated cells and paired tissue samples using Ribozol (VWR Life Science). After that, RNA samples were subjected to genomic DNA removal by incubation with DNase I (Invitrogen) at 37 °C for 2 h. Electrophoresis was performed to determine the integrity of RNAs using urea-PAGE gel. RNA purity was evaluated by checking the OD260/280 ratio of the RNA samples.

Synthesis of cDNA samples was performed using SuperScript™ III First-Strand Synthesis System (Thermo Fisher) through the following conditions: 25 °C for 5min, 52 °C for 20 min, and 85 °C for 10 min. QPCRs were then performed to measure the expression levels of circENTPD7 and PTEN mRNA with GAPDH as an internal control. The 2^-ΔΔCT^ method was used to normalize Ct values, and all qPCRs were replicated for 3 times. Primer sequences were: 5'-ATGCCAGTGATTACCTTCGT-3' (forward) and 5'-CTTCAAGCTCCCCTACTC-3' (reverse) for circENTPD7； 5'-GGACGAACTGGTGTAATGATAT-3' (forward) and 5'-TCTACTGTTTTTGTGAAGTACA-3' (reverse) for PTEN mRNA; 5'-CTCAGACACCATGGGGAAGGTG-3' (forward) and 5'-ATGATCTTGAGGCTGTTGTCAT-3' (reverse) for GAPDH mRNA.

### ELISA

Homogenates of paired tissues from the 64 NSCLC patients were prepared. PTEN ELISA Kit (Human, OKEH02377, Aviva Systems Biology) was used to determine the expression levels of PTEN. The sensitivity was 0.1 ng/mL and the detection range was 0.156 - 10 ng/mL. The levels of PTEN were normalized to ng/g tissue.

### Western blot analysis

Proteins were isolated from cells using RIPA solution (Invitrogen). Protein concentrations were measured by BCA assay (Invitrogen). After denaturation, electrophoresis (8% SDS-PAGE gel) and gel transfer to PVDF membranes were performed. Blocking of the membrane was then performed in 5% non-fat milk at room temperature for 2 h. After that, membranes were incubated with GAPDH (ab9845, Abcam) or TOB1 (ab137337, Abcam) primary antibodies at 4 ºC for at least 16 h, followed by incubation with secondary antibody of HRP Goat Anti-Rabbit (IgG) (ab6721, Abcam) at room temperature for 2 h. Signal production was performed by ECL (Sigma-Aldrich). Quantity One software (Bio-Rad) was used for data normalizations.

### CCK-8 assay

CCK-8 assay was performed using a kit from Abcam (ab228554). Cell culture was performed in a 96-well plate. The OD values were measured at 450 nm every 24 h for 4 times. Each well was added with 10% CCK-8 solution at 2 h before the measurement.

### Statistical analysis

ANOVA Tukey’s test was performed to compare multiple cell transfection groups. Paired *t* test was performed to compare paired tissues. Survival curve analysis was performed by dividing the 64 NSCLC patients into two groups (cutoff value = median expression level value). Log-rank test was used for the comparison of survival curves. *P* < 0.05 was considered statistically significant.

## Results

### Overexpression of circENTPD7 predicted poor survival of NSCLC patients

**Figure 1-:**
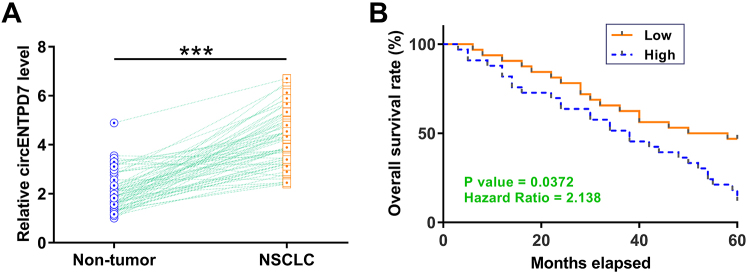
Overexpression of circENTPD7 predicted the poor survival of NSCLC patients. Expression of circENTPD7 in 64 pair tissues from the 64 NSCLC patients was determined by RT-qPCR. Average values of three technical replicates were calculated to express the data of gene expression levels in paired tissues. ***, *p* < 0.001. Survival analysis was performed through the method described in the methods section (B).

RT-qPCR results showed that circENTPD7 was significantly upregulated in NSCLC tissues compared to that in non-tumor tissues (Figure 1A, *p* < 0.001). The overall survival was significantly lower in the high circENTPD7 level group in comparison to that in the low circENTPD7 level group (Figure 1B). Therefore, overexpression of circENTPD7 may predict poor survival of NSCLC patients.

### PTEN was downregulated in NSCLC and inversely correlated with circENTPD7

**Figure 2 -:**
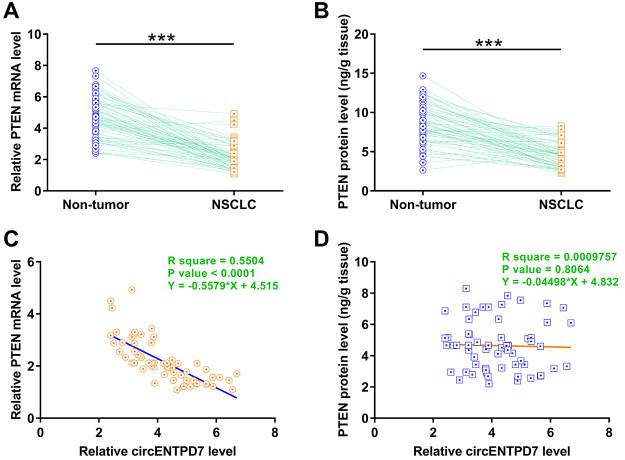
PTEN was downregulated in NSCLC and was inversely correlated with circENTPD7. Expression of PTEN mRNA (A) and protein (B) in the 64 pairs of tissues were determined by RT-qPCR and ELISA, respectively. Average values of three technical replicates were calculated to express the data of gene expression in paired tissues. ***, *p* < 0.001. Pearson’s correlation was carried out to explore the correlations between circENTPD7 and PTEN mRNA (C) or PTEN protein (D).

The expression of PTEN in the 64 pairs of tissues was determined by RT-qPCR and ELISA at mRNA and protein level, respectively. It was observed that PTEN was significantly upregulated at both mRNA (Figure 2A, *p* < 0.001) and protein (Figure 2B, *p* < 0.001) levels in NSCLC tissues. Correlation analysis showed that the expression of PTEN mRNA was not significantly correlated with circENTPD7 (Figure 2C). In contrast, the expression of PTEN protein was inversely correlated with circENTPD7 (Figure 2D). Therefore, circENTPD7 may affect the accumulation of PTEN protein.

### Overexpression of circENTPD7 decreased the expression levels of PTEN protein in NSCLC cells

**Figure 3-:**
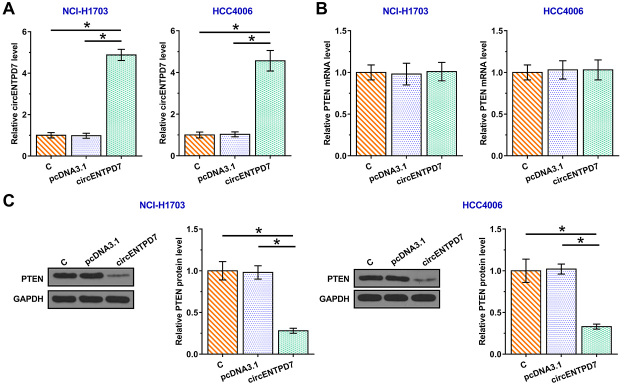
Overexpression of circENTPD7 decreased the levels of PTEN protein in NSCLC cells. To explore the effects of circENTPD7 on PTEN, NCI-H1703 and HCC4006 cells were transfected with circENTPD7 expression vector (A). The effects of circENTPD7 on PTEN mRNA (B) and PTEN protein (C) were determined by RT-qPCR and Western blot, respectively. Mean ± SD values were used to express data of three biological replicates. *, *p* < 0.05.

To explore the effects of circENTPD7 on PTEN, NCI-H1703 and HCC4006 cells were transfected with circENTPD7 expression vector (Figure 3A, *p* < 0.05). CircENTPD7 did not affect the expression of PTEN mRNA (Figure 3B), but significantly decreased the expression levels of PTEN protein (Figure 3C). Therefore, circENTPD7 may decrease the accumulation of PTEN in NSCLC cells. 

### Overexpression of circENTPD7 promoted the proliferation of NSCLC cells through PTEN

**Figure 4-:**
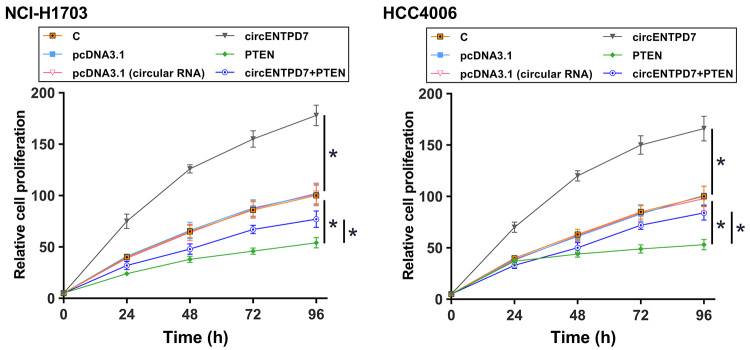
Overexpression of circENTPD7 increased NSCLC cell proliferation through PTEN. The role of circENTPD7 and PTEN in regulating the proliferation of NSCLC cells was explored by CCK-8 assay. Mean ± SD values were used to express data of three biological replicates. *, *p* < 0.05.

The role of circENTPD7 and PTEN in regulating the proliferation of NSCLC cells was explored by CCK-8 assay. It was observed that overexpression of circENTPD7 promoted the proliferation of NSCLC cells, while overexpression of PTEN decreased cell proliferation. In addition, overexpression of circENTPD7 reduced the inhibitory effects of overexpression of PTEN on cell proliferation (Figure 4, *p* < 0.05).

## Discussion

In this study we explored the interaction between circENTPD7 and PTEN in NSCLC. We found that circENTPD7 was significantly upregulated in NSCLC. In addition, overexpression of circENTPD7 reduced the accumulation of PTEN, which is the major tumor suppressive factor in cancer biology (Carnero *et al.,* 2008). 

Based on our knowledge, the function of circENTPD7 has only been explored in glioblastoma (Zhu *et al.,* 2020). It was observed that circENTPD7 was upregulated in glioblastoma and targeted ROS1 to increase the proliferation of tumors (Zhu *et al.,* 2020). This study reported the upregulation of circENTPD7 in both LUAD and LUSC, which are the two major subtypes of NSCLC. In addition, overexpression of circENTPD7 increased the proliferation of both LUAD and LUSC cells. Therefore, circENTPD7 may play an oncogenic role in NSCLC by increasing cell proliferation.

It is estimated that the 5-year-survival rate of NSCLC patients is only less than 24% after the initial diagnosis, mainly owing to the low early diagnostic rate and the lacking of effective treatment approaches for metastatic NSCLC (Wong *et al.,* 2017). In this study we proved that the high expression levels of circENTPD7 were closely correlated with the poor survival, suggesting the potential role of circENTPD7 as a prognostic biomarker for NSCLC. Therefore, measuring the expression levels of circENTPD7 in NSCLC tissues before treatment may assist the prognosis of NSCLC, which in turn guides the determination of treatments to improve patients’ survival.

PTEN plays a tumor suppressive role mainly by suppressing the PI3K/Akt pathway, which is the major cell survival pathway in cancer biology (Chalhoub and Baker, 2009). In effect, circular RNAs may also interact with PTEN to participate in cancer biology (Ma *et al.,* 2018). Circular RNAs regulate gene expression at transcription and translation levels (Lei *et al.,* 2019). In this study, we showed that circENTPD7 did not affect the expression levels of PTEN mRNA but reduced the expression levels of PTEN protein in NSCLC cells. Therefore, circENTPD7 may affect the translation or degradation of PTEN in NSCLC cells. However, more studies are needed to further explore the mechanism.

Our study characterized circENTPD7 as a novel inhibitor of PTEN translation. This novel finding may provide insights to studies of the function of circRNAs. However, our study is limited by the small sample size (n=64). Our conclusions should be verified by future studies with much bigger sample size. In addition, the in vivo interaction between circENTPD7 and PTEN should also be further verified using animal model experiments.

In conclusion, CircENTPD7 is significantly overexpressed in NSCLC. In addition, circENTPD7 may reduce the accumulation of PTEN in NSCLC cells to promote cell proliferation.
